# Evaluation of hepatic and kidney dysfunction among newly diagnosed HIV patients with viral hepatitis infection in Cape Coast, Ghana

**DOI:** 10.1186/s13104-019-4513-8

**Published:** 2019-07-31

**Authors:** Nsoh Godwin Anabire, William Jackson Tetteh, Dorcas Obiri-Yaboah, Isaac Annan, Arnold Togiwe Luuse, Paul Armah Aryee, Gideon Kofi Helegbe, Oheneba Charles Kofi Hagan, Sabastian Eliason

**Affiliations:** 1 School of Medical Sciences, University of Cape Coast, Cape Coast, Ghana; 2 Department of Biochemistry & Molecular Medicine, School of Medicine and Health Sciences, University for Development Studies, P. O. Box TL 1883, Tamale, Ghana; 3 Department of Medical Laboratory Science, School of Allied Health Sciences, University of Health and Allied Sciences, Ho, Ghana; 4 Howard Community College, 10901 Little Patuxent Parkway, Columbia, MD 21044 USA; 5 Department of Nutritional Sciences, School of Allied Health Sciences, University for Development Studies, P. O. Box TL 1883, Tamale, Ghana

**Keywords:** HIV, HBV, Clinical proxies, Hepatotoxicity, Chronic kidney disease

## Abstract

**Objective:**

HIV positive individuals infected with viral hepatitis B (HBV) or C (HCV) are at an increased risk of progression to kidney and liver failures. Therefore, prior to initiation of antiretroviral therapy, early diagnosis and initiation of appropriate treatment protocols are imperative for co-infected individuals. This study evaluated the prevalence of HBV and HCV, and extent of liver and renal dysfunction among 90 newly diagnosed HIV patients attending the Cape Coast Teaching Hospital HIV clinic.

**Results:**

Levels of alanine aminotransferase, aspartate-platelet ratio index and estimated glomerular filtration rate were used respectively to diagnose hepatotoxicity, liver fibrosis and chronic kidney disease (CKD). Association analyses were evaluated by Pearson’s Chi-square test or Fisher’s exact test and considered significant at *p* < 0.05. Using rapid diagnostic tests, 75.6% (n = 68) had HIV1 mono-infection, 24.4% (n = 22) had HIV1/HBV co-infection while 0.0% (n = 0) had HIV1/HCV co-infection. The prevalence of hepatotoxicity, liver fibrosis, and CKD were 7.8% (n = 7), 2.2% (n = 2), and 15.5% (n = 14) respectively. Similar proportions of HIV1/HBV and HIV1 were diagnosed with liver fibrosis (*p *= 0.431). In relation to hepatotoxicity Grade, a high proportion of HIV1/HBV were diagnosed with Grade 2 (*p *= 0.042). Also, severely reduced kidney function (CKD stage 4) was observed in only HIV1/HBV (n = 2, 9.1%, *p *= 0.053).

## Introduction

Human immune deficiency virus (HIV) infection remains a public health problem worldwide with about 36.9 million infections as at 2017. Sub Saharan Africa bears the greatest burden with about 25.7 million cases [1]. Generally, viral hepatitis B (HBV) and C (HCV) infections are common among people living with HIV/AIDS (PLWHA) because they tend to share the same routes of transmission [2]. Worldwide, about 5–20% of PLWHA are co-infected with HBV [3]. In Sub-Saharan Africa, the prevalence of HIV/HBV co-infection ranges between 0 and 28.4% [4, 5]. Globally, the prevalence of HIV/HCV co-infection is about 6.2% with about 2.3 million people affected [6].

The introduction of antiretroviral therapy (ART) for HIV treatment has led to reduced mortality among people living with HIV/AIDS (PLWHA) [7]. However, non-HIV related conditions such as hepatic and renal dysfunction are increasingly recognized to contribute to morbidity and mortality in HIV patients [8]. Attributive factors could be co-infection with HBV and HCV coupled with hepatotoxic/nephrotoxic impacts of ART [8, 9].

While the hepatological impact of HBV and HCV infections is well recognized, strong associations have been observed between these viral infections and renal disease [10, 11]. The pathogenesis and pathology of renal disease caused by these viruses has been extensively reviewed elsewhere [12]. Increasingly, co-infections with viral hepatitis are recognized to complicate clinical profiles [13], and among pregnant women, an increased disease burden has been reported [14, 15]. For HIV positive patients on ART, co-infection with HBV and/or HCV could exacerbate liver and kidney dysfunction. Therefore, early diagnosis of co-infected individuals and the initiation of appropriate treatment protocols are imperative. Although the World Health Organization (WHO) recommends screening of all newly diagnosed HIV individuals for HBV and HCV [16], most resource-limited countries including Ghana are yet to include this in their HIV control programmes [17]. Therefore, we undertook this pilot study to determine the prevalence of HBV and HCV and associated co-morbid conditions such as hepatic and renal dysfunction in newly diagnosed HIV infected patients attending the Cape Coast Teaching Hospital HIV clinic, prior to initiation of ART.

## Main text

### Methods

#### Study area, design and population

The study was undertaken at Cape Coast Teaching Hospital in the Cape Coast Metropolitan area of the Central Region of Ghana. This was a hospital-based retrospective cross-sectional study undertaken from January to May 2017. Newly diagnosed HIV patients who were attending the Cape Coast Teaching Hospital HIV Clinic were recruited into the study after informed consent was sought. At the clinic, patients confirmed to have HIV were assessed clinically by physicians to determine the WHO clinical stage and their suitability for treatment. The patients then undertook liver and kidney function tests, and full blood count test. HBV and HCV testing were performed during their subsequent visit prior to initiation of ART.

#### Data collection and processing

Demographic data (including age, sex, marital, occupational and educational statuses) and HIV clinical stage; which is based on the WHO clinical staging of HIV [18] were obtained from patients’ records.

Laboratory test results were retrieved from patients’ records. Full blood count data collected included hemoglobin concentration (g/dL) and platelet count (10^9^/L). Other results collected included creatinine concentration, alanine aminotransferase (ALT) concentration and aspartate aminotransferase (AST) concentration.

Hepatotoxicity was diagnosed if ALT concentrations were ≥ 50.0 U/L, and was categorized as Grades 1, 2 and 3 and 4, with ALT value of 50.0–99.9 U/L, 100.0–199.9 U/L and ≥ 200.0 U/L respectively [19]. To determine whether a patient has liver fibrosis, we calculated the aspartate-platelet ratio index using the formula: APRI = (AST/ULN) × 100)/platelet count (10^9^/L) (https://www.hepatitisc.uw.edu/page/clinical-calculators/apri). Where ULN represents the upper limit of normal range values for AST. Normal liver function was defined by APRI < 1.5, while liver fibrosis was defined by APRI > 1.5.

To determine renal function of the patients, estimated glomerular filtration rate (eGFR) was obtained using the online eGFR calculator at https://www.niddk.nih.gov/health-information/communication-programs/nkdep/laboratory-evaluation/glomerular-filtration-rate-calculators/ckd-epi-adults-conventional-unit. Parameters including serum creatinine concentration in mg/dL, race and gender were used [20]. Chronic kidney disease (CKD) stages were categorized based on the Kidney Disease Outcomes Quality Initiative classification by the National Kidney Foundation [21]. For this study, CKD was defined by CKD stages 3, 4 and 5 (with eGFR 30–59 mL/min/1.73 m^2^, 15–29 mL/min/1.73 m^2^ and < 15 mL/min/1.73 m^2^ respectively) while normal kidney function was defined by CKD stages 1 and 2 (with eGFR ≥ 90 mL/min/1.73 m^2^ and 60–89 mL/min/1.73 m^2^ respectively) [21, 22].

#### Hepatitis B and C testing

Testing for HBV infection was undertaken using the Hepatitis B combo test kit (Gemc Technology Group, Zhengzhou, Henan, China). Briefly, about 2.5 µL of capillary blood was taken from a finger prick. About 0.5 µL of the capillary blood each was spotted into wells assigned to HBsAg, HBsAb, HBeAg, HBeAb and HBcAb and a buffer solution applied immediately. HCV infection was tested using rapid anti-HCV test strip (Intec Products, Xiamen, China). For both HBV and HCV tests, the procedure, results reading and interpretations were conducted with strict adherence to the manufacturer’s protocol.

#### Statistical analysis

Data were entered into excel and the analyses done with Graphpad Prism 6 (GraphPad Software Inc., San Diego, USA) and SPSS Version 20 (IBM Corporation, Chicago, USA). Parametric variables were presented as mean and standard deviation and compared by unpaired Student t-test. Non-parametric variables were described as median and interquartile range and compared by Mann–Whitney test. Categorical variables on the other hand were presented as proportions, with 95% confidence interval and compared by Pearson’s Chi-square test or Fisher’s exact test.

### Results

#### Socio-demographic characteristics of study participants

The mean age ± SD of the 90 participants was 42.8 ± 8.2, and most (n = 63, 70.0%) were aged between 36 and 55 years (Table 1). Majority of the participants were married (n = 76, 84.4%), and most of them were females (n = 58, 64.4%) (Table 1). Although 92.2% (n = 83) of the participants had lower forms of education (none or basic form of formal education), about 94.6% (n = 85) had some form of employment (Table 1).

**Table 1:** Distribution of socio-demographic characteristics among the study participants (n = 90)

Category	Sub-category	n (%)	95% CI
Age	18–35	20 (22.2)	26.3, 31.4
	36–55	63 (70.0)	43.8, 46.5
	56+	7 (7.8)	55.5, 65.6
Sex	Female	58 (64.4)	53.9, 73.8
	Male	32 (35.6)	26.2, 46.1
Marital Status	Married	76 (84.4)	75.2, 90.7
	Widowed	2 (2.2)	0.5, 8.7
	Single	10 (11.1)	6.0, 19.6
	Divorced	1 (1.1)	0.2, 7.7
	Cohabiting	1 (1.1)	0.2, 7.7
Occupational status	Employed	85 (94.6)	88.6, 98.4
	Student/unemployed	5 (4.4)	1.6, 11.4
Highest educational level	None	50 (55.6)	45.0, 65.6
	Basic	33 (36.7)	27.2, 47.3
	Secondary	6 (6.7)	3.0, 14.2
	Tertiary	1 (1.1)	0.2, 7.7

Socio-economic determinants were presented as numbers and proportions [n (%)], with 95% confidence intervals (95% CI). None and basic: lower forms of education, secondary and tertiary: higher forms of education

#### Prevalence of viral hepatitis B or C in newly diagnosed HIV infections

All the 90 participants tested positive for HIV1 with none testing positive for HIV2. Among the participants, 24.4% (n = 22) tested positive for both HBsAg and HBcAb, 5.6% (n = 5) tested positive for HBsAb, 2.2% (n = 2) tested positive for HBeAg and 8.9% (n = 8) tested positive for HBeAb. The prevalence of HIV1 mono-infection was 75.6% (n = 68) and that of HIV1/HBV co-infection was 24.4% (n = 22). We did not record any HCV infection.

#### Diagnosis of HIV clinical stages

Majority of the participants, 71.1% (n = 64, 95% CI 60.7–79.7), were diagnosed with HIV stage 1. Of the remaining participants, 14.4% (n = 13, 95% CI 8.5–23.5) had HIV stage 2, 7.8% (n = 7, 95% CI 3.7–15.6) had HIV stage 3 and 6.7% (n = 6, 95% CI 3.0–14.2) had HIV stage 4.

#### Comparison of socio-economic variables between the different groups of HIV infections

The mean age between the HIV1 mono-infection and HIV1/HBV co-infection groups was similar (*p *= 0.984, Table 2). Also, infection with either HIV1 or HIV1/HBV was independent of age group (*p *= 0.808), gender (*p *= 0.927), marital status (*p *= 0.819), level of education (*p *= 0.480) and occupational status (*p *= 0.569) (Table 2).

**Table 2:** Association between socio-demographic factors and HIV infection type

Characteristics	HIV1 mono-infection (n = 68)	HIV1/HBV co-infection (n = 22)	*p*-value
Age, mean ± SD	42.9 ± 10.4	42.8 ± 8.2	0.984
Age group, n (%)			
18–35	15 (16.7)	5 (5.6)	0.808
36–55	47 (52.2)	16 (17.8)	
56+	6 (6.7)	1 (1.1)	
Sex, n (%)			
Female	44 (48.9)	14 (15.6)	0.927
Male	24 (26.7)	8 (8.9)	
Marital status, n (%)			
Married	56 (62.2)	20 (22.2)	0.819
Widowed	2 (2.2)	0 (0.0)	
Single	8 (8.9)	2 (2.2)	
Divorced	1 (1.1)	0 (0.0)	
Cohabiting	1 (1.1)	0 (0.0)	
Occupational status, n (%)			
Employed	64 (71.2)	22 (24.4)	0.569
Student/unemployed	4 (4.4)	0 (0.0)	
Highest educational level, n (%)			
None	24 (26.7)	9 (10.0)	0.480
Basic	37 (41.1)	13 (14.4)	
Secondary	6 (6.7)	0 0.0)	
Tertiary	1 (1.1)	0 (0.0)	

Unpaired t-test was used to compare the exact ages between the mono-infected and co-infected participants. Where SD = standard deviation of the mean ages. Association analyses between infection type and the socio-economic determinants were done using Pearson’s Chi-square test or Fisher’s exact test. A two-tailed *p*-value was considered significant at *p *< 0.05

#### Prevalence of hepatic damage, liver fibrosis and CKD among the newly diagnosed HIV infections

The levels of hematological indices [Hb (*p* = 0.917) and PLT (*p* = 0.460)], liver chemistry [ALT (*p* = 0.821) and AST (*p* = 0.123)] and renal chemistry [creatinine (*p* = 0.351)] tests were observed to be similar in both HIV1 and HIV1/HBV groups (Table 3). The prevalence of hepatotoxicity, liver fibrosis, and chronic kidney disease (CKD) were 7.8% (n = 7), 2.2% (n = 2), and 15.5% (n = 14) respectively. Similar proportions of the HIV1 and HIV1/HBV groups were diagnosed with hepatotoxicity (*p* = 0.355), liver fibrosis (*p *= 0.431) and CKD (*p* = 0.696) (Table 3). In relation to the hepatotoxicity Grade, a high proportion of those with HIV1/HBV were diagnosed with Grade 2 (*p *= 0.042, Table 3). Also, severely reduced kidney function (CKD stage 4) was recorded in only those with HIV1/HBV (n = 2, 9.1%), and such an observation was nearly significant (*p* = 0.053, Table 3). That notwithstanding, no differences in HIV clinical stages were observed between the two groups (*p* = 0.86, Table 3).

**Table 3:** Association between HIV clinical data and HIV infection type

Clinical data	Description	HIV1 mono-infection (n = 68)	HIV1/HBV co-infection (n = 22)	*p*-value
		Median (IQR)		
Hb (g/dL)		10. 0 (8.5–11.8)	10.4 (8.8–11.4)	0.917
PLT (10^9^/L)		208 (165.0–245.0)	230 (167.3–256.3)	0.460
ALT (U/L)		28.1(18.1–34.7)	25.6 (17.3–45.3)	0.821
AST (U/L)		30.6 (23.8–45.0)	33.0 (27.5–58.9)	0.123
Creatinine (mg/dL)		80.0 (65.1–106.2)	89.8 (62.0–118.2)	0.351
	ALT (U/L)	Hepatotoxicity, n (%)		
Absent	< 50.0	64 (91.4)	19 (86.4)	0.355
Present	≥ 50.0	4 (5.9)	3 (13.6)	
Grade 1	50.0–99.9	4 (5.9)	1 (4.5)	0.042
Grade 2	100.0–199.9	0 (0.0)	2 (9.1)	
Grade 3 and 4	≥ 200.0	0 (0.0)	0 (0.0)	
	APRI	Liver fibrosis, n (%)		
Absent	< 1.5	67 (98.5)	21 (95.5)	0.431
Present	> 1.5	1 (1.5)	1 (4.5)	
	eGFR (mL/min/1.73 m^2^)	CKD, n (%)		
Absent	≥ 60	58 (85.3)	18 (81.8)	0.696
Present	< 60	10 (14.7)	4 (18.2)	
Stage 1	≥ 90 Normal GFR	40 (58.8)	10 (45.5)	0.053
Stage 2	60–89 Mildly reduced GFR	18(26.5)	8 (36.4)	
Stage 3	30–59 Moderately reduced GFR	10 (14.7)	2 (9.1)	
Stage 4	15–29 Severely reduced GFR	0 (0.0)	2 (9.1)	
		n (%)		
HIV stages	Stage 1	47 (52.2)	17 (18.9)	0.86
	Stage 2	10 (11.1)	3 (3.3)	
	Stage 3	6 (6.7)	1 (1.1)	
	Stage 4	5 (5.6)	1 (1.1)	

Mann–Whitney test was used to compare levels of Hb, PLT, ALT and AST. Where IQR = interquartile range. Association analyses of infection type with hepatotoxicity, liver fibrosis CKD or HIV stages were done using Pearson’s Chi-square test or Fisher’s exact test. A two-tailed *p*-value was considered significant at *p *< 0.05

### Discussion

HIV infection complicated by HBV and HCV infection increases the risk of morbidity and mortality compared with HIV mono-infection. Because treatment increases the survival of co-infected individuals compared with the untreated, it is recommended to routinely check for HBV and HCV status of all newly diagnosed patients. However, in most resource-limited countries including Ghana, this recommendation is yet to be included in HIV control programmes. In this pilot study, all newly diagnosed patients were HIV1 positive with none testing positive for HIV2 infection. In Ghana, 98.5% of HIV infections are due to the type 1 mono-infection, with the rest being dual type 1 and 2 infections [23]. The 22.4% prevalence of HIV/HBV co-infection falls within the range of 0 to 28.4% reported in sub Saharan Africa [7]. This prevalence was as expected, as most HBV acquisition in Sub-Sahara Africa, including Ghana, is before age 6 years, thus, HIV/HBV co-infection usually mirrors the national HBV prevalence [5, 24]. We did not record any case of HIV/HCV co-infection, probably because, the prevalence of this co-infection tends to be lower in sub Saharan Africa; where intravenous drug injection is less prevalent [6]. However, Ntiamoah in 2015 reported an HIV/HCV prevalence in Ghanaian pregnant women as 4.1% [25] whereas Brandful et al. reported a prevalence of 8.2% among patients attending sexually transmitted disease clinics in 1999 [26].

To determine the impact of HIV/HBV co-infection on liver morbidity such as hepatotoxicity and liver fibrosis in our study participants, we utilized surrogates such ALT, AST, APRI and degree of hepatotoxicity. We observed no significant differences in these surrogates, that notwithstanding, studies in Tanzania reported a significantly higher AST and ALT concentration in HIV/HBV co-infected individuals compared with HIV mono-infected [27, 28]. Though several non-invasive measurements exist for diagnosing liver fibrosis [29, 30], the APRI is recommended by the WHO for assessing the presence of liver fibrosis in the absence liver biopsy [17, 31]. Using > 1.5% APRI proxy, the study reports a prevalence of 2.2% of liver fibrosis among the participants, which is lower compared with high levels of liver fibrosis reported elsewhere in Sub Saharan Africa [27, 32, 33]. Even though HIV/HBV co-infected patients are at an increased risk of developing liver fibrosis compared with HIV mono-infected individual [34], our findings revealed that co-infection was independent of liver fibrosis. The prevalence of hepatotoxicity was 7.8%, which compares with the 6.5% reported in Ethiopia [35], however, the higher rate in this study could be due to the presence of co-infection with HBV.

The use of tenofovir and/or lamivudine in ART combination for treating HIV complicated with HBV co-infection has been recommended irrespective of the immune state of the patients [36]. However, these drugs have been found to increase the risk of developing nephrotoxicity in these patients [35, 37, 38]. Therefore, assessment of the renal function prior to treatment has been recommended [16, 37]. The 15.5% prevalence of CKD in the newly diagnosed compares with a high rate of 22.9% recorded in Nigeria [39]. kidney failure may result from HIV-associated nephropathy, which is common in the Africans with contributory factors such as co-infection with HBV or HCV [40, 41]. Therefore, our finding of higher proportions of HIV1/HBV co-infected individuals with severe forms of CKD (CKD 4) buttresses such findings.

### Conclusion

In summary, the study observed that, prior to initiation of ART, severe forms of hepatotoxicity (hepatotoxicity Grade 2) and CKD (CKD stage 4) were common among HIV1/HBV co-infections. The findings buttress the need for resource-limited countries to routinely screen for hepatitis B at HIV clinics, so as to initiate appropriate treatment protocols.

## Limitation

The sample size for the study was small, thus, future studies with a larger sample size is recommended to substantiate our findings. The underlying aetiology of liver and kidney dysfunction was unknown for the participants. Also, the study could not diagnose CKD in participants with eGFR values > 60, since urine albumin, an alternative mean value for determining CKD was not measured.

## Data Availability

The datasets supporting the findings of this article are available in this manuscript.

## Abbreviations

HBV: hepatitis B virus
HCV: hepatitis C virus
CKD: chronic kidney disease
PLWHA: people living with HIV/AIDS
Hb: hemoglobin
PLT: platelets
ALT: alanine aminotransferase
AST: aspartate aminotransferase
ULN: upper limit of normal
eGFR: estimated glomerular filtration rate
APRI: aspartate aminotransferase to platelet ratio index
WHO: World Health Organization

## References

[1] World Health Organization. HIV/AIDS: key facts. 2018. https://www.who.int/en/news-room/fact-sheets/detail/hiv-aids. Accessed 17 Dec 2018.

[2] Alter MJ (2006). Epidemiology of viral hepatitis and HIV co-infection. J Hepatol.

[3] World Health Organization. HIV and hepatitis coinfections. 2019. https://www.who.int/hiv/topics/hepatitis/en/. Accessed 16 July 2019.

[4] Stabinski L, O’connor S, Barnhart M, Kahn RJ, Hamm TE (2015). Prevalence of HIV and hepatitis B virus co-infection in sub-Saharan Africa and the potential impact and program feasibility of hepatitis B surface antigen screening in resource-limited settings. JAIDS.

[5] Hoffmann CJ, Thio CL (2007). Clinical implications of HIV and hepatitis B co-infection in Asia and Africa. Lancet Infect Dis.

[6] Platt L, Easterbrook P, Gower E, McDonald B, Sabin K, McGowan C, Yanny I, Razavi H, Vickerman P (2016). Prevalence and burden of HCV co-infection in people living with HIV: a global systematic review and meta-analysis. Lancet Infect Dis.

[7] Matthews PC, Geretti AM, Goulder PJ, Klenerman P (2014). Epidemiology and impact of HIV coinfection with hepatitis B and hepatitis C viruses in Sub-Saharan Africa. J Clin Virol.

[8] Goehringer F, Bonnet F, Salmon D, Cacoub P, Paye A, Chêne G, Morlat P, May T, ANRS EN20 Mortalité 2010 Study Group (2017). Causes of death in HIV-infected individuals with immunovirologic success in a national prospective survey. AIDS Res Hum Retroviruses..

[9] Singh KP, Crane M, Audsley J, Avihingsanon A, Sasadeusz J, Lewin SR (2017). HIV-hepatitis B virus coinfection: epidemiology, pathogenesis, and treatment. AIDS.

[10] Hong YS, Ryu S, Chang Y, Caínzos-Achirica M, Kwon MJ, Zhao D, Shafi T, Lazo M, Pastor-Barriuso R, Shin H, Cho J (2018). Hepatitis B virus infection and development of chronic kidney disease: a cohort study. BMC Nephrol.

[11] Cacoub P, Desbois AC, Isnard-Bagnis C, Rocatello D, Ferri C (2016). Hepatitis C virus infection and chronic kidney disease: time for reappraisal. J Hepatol.

[12] Chacko EC, Surrun SK, Sani TM, Pappachan JM (2010). Chronic viral hepatitis and chronic kidney disease. Postgrad Med J.

[13] Anabire NG, Aryee PA, Abdul-Karim A, Quaye O, Awandare GA, Helegbe GK (2019). Impact of malaria and hepatitis B co-infection on clinical and cytokine profiles among pregnant women. PLoS ONE.

[14] Helegbe GK, Aryee PA, Mohammed BS, Wemakor A, Kolbila D, Abubakari AW, Askanda S, Alhassan R, Barnie C, Donkoh AA, Ofosu E (2018). Seroprevalence of malaria and hepatitis B coinfection among pregnant women in tamale metropolis of Ghana: a cross-sectional study. Can J Infect Dis Med Microbiol.

[15] Anabire NG, Aryee PA, Abdul-Karim A, Abdulai IB, Quaye O, Awandare GA, Helegbe GK (2019). Prevalence of malaria and hepatitis B among pregnant women in Northern Ghana: comparing RDTs with PCR. PLoS ONE.

[16] World Health Organization. Guidelines for the prevention, care and treatment of persons with chronic hepatitis B infection. 2015. http://www.who.int/hiv/pub/hepatitis/hepatitis-b-guidelines/en/. Accessed 16 Aug 2015. 26225396

[17] Coffie PA, Egger M, Vinikoor MJ, Zannou M, Diero L, Patassi A, Kuniholm MH, Seydi M, Bado G, Ocama P, Andersson MI (2017). Trends in hepatitis B virus testing practices and management in HIV clinics across sub-saharan Africa. BMC Infect Dis.

[18] World Health Organization. Clinical staging of HIV disease in adults, adolescents and children. 2016. https://www.ncbi.nlm.nih.gov/books/NBK374293/. Accessed 16 May 2018.

[19] US Department of Health and Human Services. National Institute of Allergy and Infectious Diseases, Division of AIDS. Division of AIDS (DAIDS) table for grading the severity of adult and pediatric adverse events, version 2.0. Division of AIDS National Institute of Allergy and Infectious Diseases, National Institute of Haelth and Human Services. 2014.

[20] Levey AS, Stevens LA, Schmid CH, Zhang YL, Castro AF, Feldman HI, Kusek JW, Eggers P, Van Lente F, Greene T, Coresh J (2009). A new equation to estimate glomerular filtration rate. Ann Intern Med.

[21] Levey AS, Coresh J, Bolton K, Culleton B, Harvey KS, Ikizler TA, Johnson CA, Kausz A, Kimmel PL, Kusek J, Levin A (2002). K/DOQI clinical practice guidelines for chronic kidney disease: evaluation, classification, and stratification. Am J Kidney Dis.

[22] Hogan M (2009). KDIGO conference proposes changes to CKD classification, but not to the definition. Nephrol Times..

[23] Ghana AIDS Commission. Summary of the 2016 HIV sentinel survey report. 2017. http://www.ghanaids.gov.gh/gac1/aids_info.php. Accessed 28 Nov 2018.

[24] Martinson FE, Weigle KA, Royce RA, Weber DJ, Suchindran CM, Lemon SM (1998). Risk factors for horizontal transmission of hepatitis B virus in a rural district in Ghana. Am J Epidemiol.

[25] Ntiamoah P. The prevalence of hepatitis B and/or hepatitis C virus (HBV and/or HCV) co-infection among HIV-infected pregnant women (Doctoral dissertation). http://ir.knust.edu.gh/bitstream/123456789/8620/1/Ntiamoah%27sResearchWork-combined.pdf.

[26] Brandful JA, Apeagyei FA, Ampofo WK, Adu-Sarkodie Y, Ansah JE, Nuvor V, Aidoo S, Ishikawa K, Sata T, Yamamoto N, Yamazaki S (1999). Relationship between immunoclinical status and prevalence of viral sexually transmitted diseases among human immunodeficiency virus-1 seropositive patients in Ghana. Viral Immunol.

[27] Hawkins C, Christian B, Fabian E, Macha I, Gawile C, Mpangala S, Ulenga N, Thio CL, Ammerman LR, Mugusi F, Fawzi W (2017). Brief report: HIV/HBV coinfection is a significant risk factor for liver fibrosis in Tanzanian HIV-infected adults. JAIDS.

[28] Kilonzo SB, Gunda DW, Kashasha F, Mpondo BC (2017). Liver fibrosis and hepatitis B coinfection among ART naive HIV-infected patients at a tertiary level hospital in northwestern Tanzania: a cross-sectional study. J Trop Med.

[29] Bottero J, Lacombe K, Guéchot J, Serfaty L, Miailhes P, Bonnard P, Wendum D, Molina JM, Lascoux-Combe C, Girard PM (2009). Performance of 11 biomarkers for liver fibrosis assessment in HIV/HBV co-infected patients. J Hepatol.

[30] DallaPiazza M, Amorosa VK, Localio R, Kostman JR, Re VL (2010). Prevalence and risk factors for significant liver fibrosis among HIV-monoinfected patients. BMC Infect Dis.

[31] Vinikoor MJ, Mulenga L, Siyunda A, Musukuma K, Chilengi R, Moore CB, Chi BH, Davies MA, Egger M, Wandeler G, International Epidemiologic Databases to Evaluate AIDS in Southern Africa (IeDEA-SA) (2016). Association between hepatitis B co-infection and elevated liver stiffness among HIV-infected adults in Lusaka, Zambia. Trop Med Int Health.

[32] Ramírez-Mena Adrià, Glass Tracy R., Winter Annja, Kimera Namvua, Ntamatungiro Alex, Hatz Christoph, Tanner Marcel, Battegay Manuel, Furrer Hansjakob, Wandeler Gilles, Letang Emilio (2016). Prevalence and Outcomes of Hepatitis B Coinfection and Associated Liver Disease Among Antiretroviral Therapy-Naive Individuals in a Rural Tanzanian Human Immunodeficiency Virus Cohort. Open Forum Infectious Diseases.

[33] Stabinski L, Reynolds SJ, Ocama P, Laeyendecker O, Boaz I, Ndyanabo A, Kiggundu V, Gray RH, Wawer M, Thio C, Thomas DL (2011). High prevalence of liver fibrosis associated with HIV infection: a cross-sectional study in rural Rakai, Uganda. Antiviral Ther.

[34] Ioannou GN, Bryson CL, Weiss NS, Miller R, Scott JD, Boyko EJ (2013). The prevalence of cirrhosis and hepatocellular carcinoma in patients with human immunodeficiency virus infection. Hepatology.

[35] Baynes HW, Tegene B, Gebremichael M, Birhane G, Kedir W, Biadgo B (2017). Assessment of the effect of antiretroviral therapy on renal and liver functions among HIV-infected patients: a retrospective study. HIV/AIDS.

[36] Terrault NA, Lok AS, McMahon BJ, Chang KM, Hwang JP, Jonas MM, Brown RS, Bzowej NH, Wong JB (2018). Update on prevention, diagnosis, and treatment of chronic hepatitis B: AASLD 2018 hepatitis B guidance. Hepatology.

[37] Herlitz LC, Mohan S, Stokes MB, Radhakrishnan J, D’Agati VD, Markowitz GS (2010). Tenofovir nephrotoxicity: acute tubular necrosis with distinctive clinical, pathological, and mitochondrial abnormalities. Kidney Int.

[38] Jotwani V, Atta MG, Estrella MM (2017). Kidney disease in HIV: moving beyond HIV-associated nephropathy. J Am Soc Nephrol.

[39] Anyabolu EN, Chukwuonye II, Arodiwe E, Ijoma CK, Ulasi I (2016). Prevalence and predictors of chronic kidney disease in newly diagnosed human immunodeficiency virus patients in Owerri, Nigeria. Indian J Nephrol.

[40] Naicker S, Rahmania S, Kopp JB (2015). HIV and chronic kidney disease. Clin Nephrol.

[41] Ugiagbe RA, Eze EU (2011). Effect of anemia on hepatotoxicity of HAART in HIV patients in Benin city. Niger Med J.

